# Synthesis and investigation of phosphorus-free ionic liquids as multifunctional lubricating additives†

**DOI:** 10.1039/d2ra04006a

**Published:** 2022-09-05

**Authors:** Huaigang Su, Qin Zhao, Yunlong Chen, Qilong Zhao, Cheng Jiang, Wenjing Lou

**Affiliations:** State Key Laboratory of Solid Lubrication, Lanzhou Institute of Chemical Physics, Chinese Academy of Sciences Lanzhou 730000 P. R. China wjlou@licp.cas.cn jiangcheng@licp.cas.cn; University of Chinese Academy of Sciences Beijing 100049 P. R. China; Qingdao Center of Resource Chemistry and New Materials Qingdao 266100 P. R. China

## Abstract

Ionic liquids (ILs) have been extensively studied as lubricants or lubricant additives for two decades by virtue of their unique physical and chemical properties. Here, two phosphorus-free multifunctional protic ILs were synthesized by reacting oleic acid and a dimer acid with an alkyl aromatic amine. Both were completely miscible in various base fluids like mineral oil, polyether, synthetic ester, and polyalpha olefin. Furthermore, no corrosion towards copper strips was found for these additives due to the absence of phosphorus, halogen, and sulfur in their molecular structures. Tribological tests found that they could significantly improve the tribological performance of base oil in a wide range of test conditions. Additionally, due to the presence of an alkyl diphenylamine group, they could considerably enhance the oxidative stability of the base oil. Overall, the facile preparation approach, good solubility, low corrosion, and excellent tribological behavior and antioxidation property make them suitable as multifunctional additives in various lubricants.

## Abstract 2

Two phosphorus-free multifunctional protic ionic liquids were devolpoed for lubricating additives. The low corrosion, excellent tribological behavior and antioxidation property make them suitable as multifunctional additives in various lubricants.

## Graphical abstract

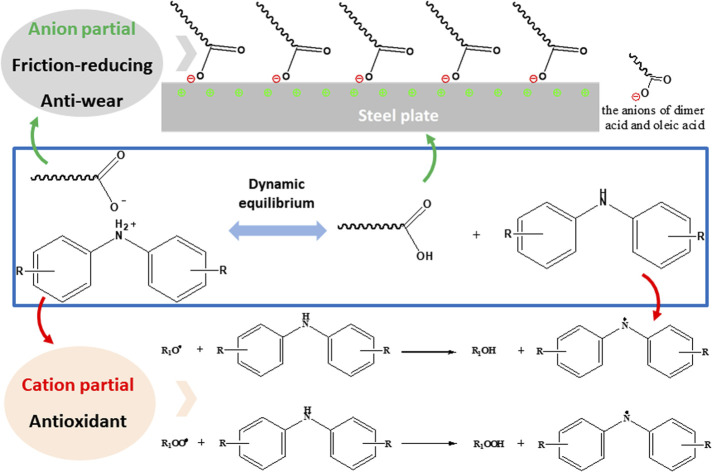

## 1. Introduction

Lubricating additives are used to impart special properties to base oils which could improve the overall performance of lubricants to tolerate various severe working conditions.^1,2^ There are many kinds of lubricant additives, including anti-wear additives, extreme-pressure additives, anti-oxidation additives, viscosity control additives, deposit control additives, anti-corrosion additives, anti-foaming additives, and so on.^3^ Zinc dialkyl-dithiophosphates (ZDDPs) were found to behave in a triple role as anti-wear additives, anti-corrosion additives and additional anti-oxidation additives.^4^ However, the phosphorus in ZDDPs could deactivate poison the catalysts used for exhaust gas treatment, and the zinc participates the formation of dust. Recently, strict environmental regulations have promoted a reduction in the use of ZDDP as an oil additive.^5^ Therefore, a great deal of research was focused on finding new additives as replacements of ZDDPs.^6,7^

Ionic liquids (ILs) have been extensively studied as neat lubricants or lubricant additives for two decades since their first reported as lubricants in 2001 by Liu and coauthors.^8^ ILs exhibit many unique physical and chemical properties such as low flammability, negligible volatility, high thermal stability, and excellent tribological performance, which are the desired properties for lubricants.^9^ Also, their high design ability of molecular structure and the diverse ranges of cations and anions provide immense potential to design task-specific ionic liquids for lubrication applications and were considered as potential candidates to replace ZDDPs. However, high cost, poor miscibility with traditional lubricating oils, poor thermal oxidation, and corrosion towards friction pairs are the major technical barriers to their application.^10,11^ Recently, many attempts have been made to solve the above obstacles. Qu reported a series of oil-soluble ILs which can be used as lubricant additives and the cost was reduced to a large extent.^12–14^ Cai and coauthors synthesized imidazolium ILs, and incorporated sterically hindered phenol and benzotriazole groups into their molecular structures, these ILs showed excellent anti-oxidation and anti-corrosion properties.^15^ Our recent work reported a multifunctional protic IL which was effective friction-reducing and anti-wear agent in polyalpha olefin (PAO4), in addition, it also exhibited excellent antioxidant properties due to the presence of diphenylamine group.^16^ However, in order to achieve better tribological properties, most of the previously reported ILs reported contained phosphorus, halogen, or sulfur elements which could be harmful to the environment.^17–19^ Fatty acids are the renewable, green, and sustainable source, and have been used to reduce the friction coefficient and synthesize protic ionic liquids in earlier works.^20,21^ However, these works focus on the tribological performances of ILs, few researchers have focused on the development of phosphorus and halogen-free ILs as multifunctional lubricant additives.

In this work, two multifunctional ILs were synthesized *via* neutralizing nonylated diphenylamine (NDPA) with oleic acid and dimer acid, respectively. Viscosity, corrosion, thermal stability, and oil solubility of ILs as lubricant additives for PAO4 were investigated. The tribological behaviors of the synthesized ILs with PAO4 blends under different lubrication conditions were carried out on SRV tester and the oxidation stability of PAO4 with ILs was also studied by the pressure differential scanning calorimeter (PDSC).

## 2. Experiment

### 2.1 Materials

NDPA with a trade name of IRGANOX L 67 was obtained from BASF Company Ltd. Dimer acid (98 wt%) was obtained from Jiangxi Eturk Industrial Co., Ltd. Oleic acid was purchased from Sinopharm Chemical Reagent Co., Ltd. PAO4, synthetic ester (5750), oil soluble polyalkylene glycol (OSP 32), and mineral oil (150SN) were obtained from Exxon Mobil, Croda lubricant, Dow Chemical, and China Petroleum & Chemical Corporation respectively.

### 2.2 Synthesis of ILs

**Fig. 1:**
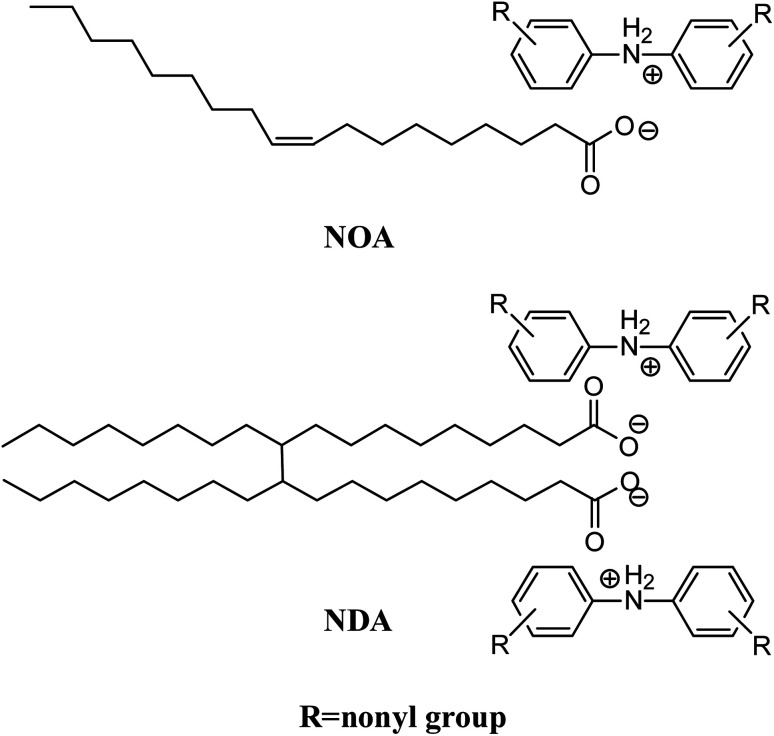
Schematic molecular structures of NOA and NDA ILs.

In a typical synthesis reaction, a mixture of an equimolar quantity of oleic acid and nonylated diphenylamine was magnetically stirred at 80 °C for 8 hours to obtain the dark brown ionic liquid (NOA). Dimer acid and nonylated diphenylamine were mixed at a molar ratio of 1 : 2 and stirred at 80 °C for 8 hours. After cooling to room temperature, a viscous dark brown ionic liquid (NDA) was obtained. Schematic molecular structures of NOA and NDA ILs were shown in Fig. 1. The syntheses of NOA and NDA were confirmed by ^1^H-NMR compared with starting materials, as shown in Fig. S1 and S2 (ESI†). The analysis results of data are also listed in ESI.†

### 2.3 FTIR analysis

Fourier transformation infrared spectroscopy (FTIR) of reactants and ILs were conducted on a Bruker Tensor 27 FTIR spectrometer in the range of 4000–600 cm^−1^.

### 2.4 Thermal analysis

Thermogravimetric analysis of ILs was measured on a thermogravimetry-differential scanning calorimetry instrument (TG-DSC, NETZSCH, STA 449, Bavarian, Germany) with a temperature range from 40 °C to 600 °C and a heat rate of 10 °C min^−1^ in air.

### 2.5 Viscosity

Kinematic viscosity at 40 and 100 °C were measured on a on a Stabinger SVM 3000 (Anton Paar GmbH, Graz, Austria) according to ASTM D7042. Viscosity index (VI) was calculated from kinematic viscosity at 40 and 100 °C according to ASTM D2270.

### 2.6 Tribological test

The friction tests were performed with a ball-on disc configuration on a oscillating reciprocating friction and wear tester (SRV, Optimol, Germany). The fixed lower specimens was AISI52100 steel disc (60–64 Rockwell hardness C (HRC), *ø* 24.00 × 7.9 mm). An AISI52100 steel ball with a diameter of 10 mm and a hardness of 58–62 HRC was rubbing against the disc. The relative humidity was 28–45%. Every friction test was repeated at least three times to guarantee accuracy. The morphology of the rubbing surfaces on discs was analyzed by the scanning electron microscopy (SEM, Carl Zeiss, Germany). To further characterize the lubrication mechanism, X-ray photoelectron spectroscopy (XPS, ThermoFisher ESCALAB 250, USA) was applied to study the surface chemical elements of wear scars after SRV test. XPS was carried with an Al Kα X-ray radiation of 1486.6 eV radiation in ultrahigh vacuum (10^−7^ Pa) and the C1s peak at 284.8 eV as a binding energy calibration.

### 2.7 Oxidation test

Anti-oxidation performances of PAO4, ILs and PAO4 blends were measured with pressurized differential scanning calorimetry (PDSC, NETZSCH DSC 204HP, Bavarian, Germany) according to ASTM D6186. 3.0 ± 0.2 mg sample was placed in a new sample pan and heated from 40 °C to 180 °C with a heating rate of 100 °C min^−1^. Then, allow the samples to equilibrate at the 180 °C for 2 min. The oxygen was added in until the pressure was achieved at 3.5 MPa ± 0.2 MPa, and it required about 2 min to reach the maximum pressure. When the pressure was reached equilibrium, the cell purge rate adjusted and maintained at 100 mL min^−1^ ± 10 mL min^−1^. Oxidation induction time (OIT) was measured from the oxygen added to an exothermic peak of oxidation appeared.

## 3. Result and discussion

### 3.1 FTIR analysis

**Fig. 2:**
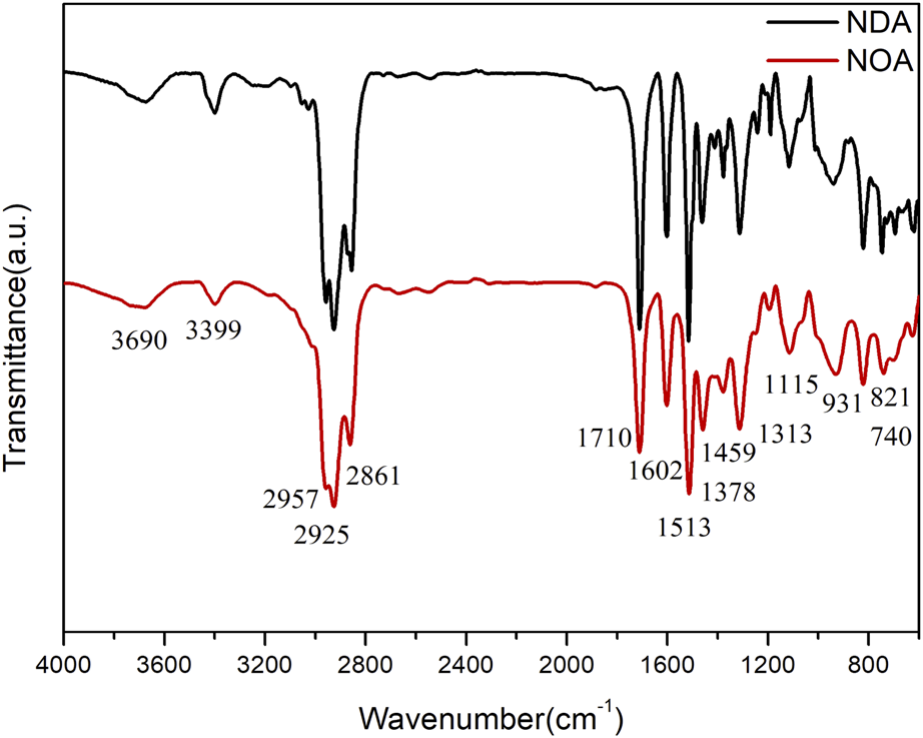
FTIR spectra of NOA and NDA.

FTIR spectra of NOA and NDA were presented in Fig. 2. The band at 3399 cm^−1^ is assigned to the N–H stretching band of nonylated diphenylamine. The strong and broad vibrational modes in the range of 3000–2800 cm^−1^ were assigned to methylene and methyl asymmetric and symmetric stretches of the long alkyl chains of the NOA and NDA ILs. The peaks at 1459 and 1378 cm^−1^ further confirmed existence of the long alkyl chains of the NOA and NDA ILs.^22^ The peak at 1602 cm^−1^ was ascribed to the asymmetrical stretching vibration of –COO^−^ from anions of NOA and NDA, indicating the neutralization reaction of fatty acid and NDPA. The characteristic peaks of the benzene ring in L67 were reflected at 1607, 1513, 821 and 740 cm^−1^. There is a broad peak at 3690 cm^−1^ corresponding to the O–H stretching band of oleic acid and dimer acid. Meanwhile, the strong stretching peak at 1710 cm^−1^ further indicated the presence of the carboxyl groups.^21^ Protic ILs NOA and NDA were synthesized by transferring a proton from acid to amine. This process involves a dynamic equilibrium between ILs and the free neutral acid and base species.^23^ So the characteristic peaks of carboxyl groups will appear in the FTIR spectrum.

### 3.2 Physicochemical property

**Fig. 3:**
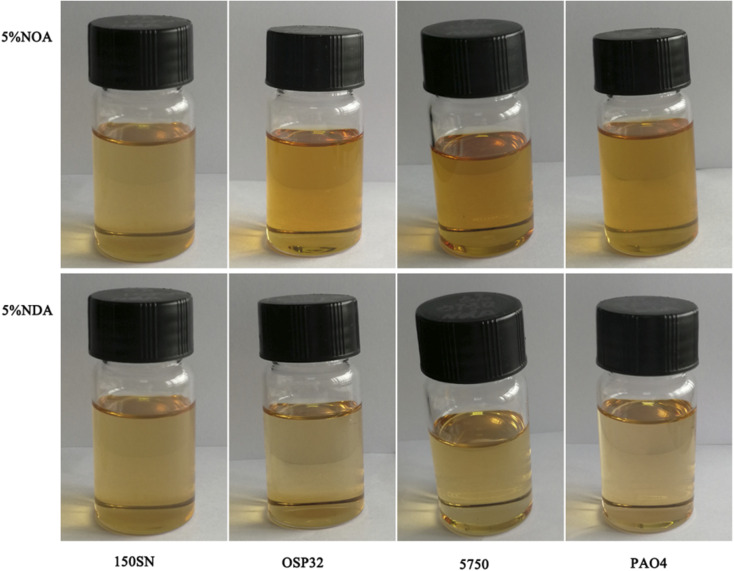
Solubility of ILs in different kind of base oils at room temperature.

Unlike most of the traditional ILs, both NOA and NDA were found to be fully miscible in a variety of lubricating base oils, such as mineral oil (150 SN), polyether (OSP 32), synthetic ester (5750), and polyalpha olefin (PAO4). Fig. 3 displayed the images of four base oils mixed with 5 wt% of ILs. All the mixtures appear clear and without any clouds or phase separation at room temperature. It's worth to mention that even after six months of storage, the mixture remained clear and without any phase separation. The exceptional oil solubility of NOA and NDA is attributed to their long hydrocarbon chain of cation and anion which could generate high steric hindrance and effectively reduce the interactions between cation and anion. A higher steric hindrance indicates a lower polarity of the ILs which enhances ILs's solubility with tradition less polarity base oils.^23^

Kinematic viscosities, viscosity index, corrosion performance, and thermal stability of different lubricants were summarized in Table 1. NDA is much more viscous than NOA due to its relatively longer alkyl chains of anions. It is reported that increasing the length of alkyl chains improves the viscosity due to the stronger van der Waals interactions.^24^ The kinematic viscosities at 40 °C of NOA and NDA blends increased slightly with the increase of additive concentration. The kinematic viscosities and viscosity index of NOA and NDA blends were similar to pure PAO4, demonstrating that these ILs have a negligible effect on the viscosity properties of base oil.

**Table 1:** Typical physicochemical properties of different lubricants

Sample	Kinematic viscosity		Viscosity index	Corrosion grade	*T*_d_
	40 °C mm^−2^ s^−1^	100 °C mm^−2^ s^−1^			
NOA	121.8	7.900	70	1b	254.7
NDA	1171	35.36	25	1b	275.5
PAO4	18.43	4.076	122	1b	253.6
1% NOA	18.52	4.120	126	1a	258.0
2% NOA	18.67	4.120	124	1a	276.5
3% NOA	18.80	4.153	125	1a	278.5
4% NOA	19.00	4.114	118	1a	280.3
5% NOA	19.09	4.164	122	1a	274.7
1% NDA	18.73	4.113	122	1a	253.7
2% NDA	19.06	4.172	123	1a	261.0
3% NDA	19.52	4.244	124	1a	272.9
4% NDA	20.22	4.326	123	1a	276.0
5% NDA	20.69	4.344	119	1a	276.4

Thermal stabilities of ILs and their blends were investigated under air atmosphere with a temperature range from 40 °C to 600 °C. The thermogravimetric results revealed that NOA, NDA, and the PAO4 blends possess good thermal stability with the temperature of decomposition (*T*_d_) results between 258 °C to 280 °C. As shown in Table 1, the *T*_d_ increased with the increased concentration of NOA, and PAO4 with 4% NOA blend showed the highest *T*_d_, which was 26.7 °C higher than PAO4. The *T*_d_ value of PAO4 with 4% NDA was close to 5% NDA, and *T*_d_ trend of PAO4 with NDA blends was similar to NOA blends. The above results indicated that NOA and NDA could significantly improve the thermal stability of PAO4. Copper strip corrosion tests were carried out to evaluate the corrosion properties of NOA and NDA. It was found that all the copper strips showed no visible color change after immersing in different lubricants for 3 hours at 100 °C, indicating that non-corrosion properties of these ILs. The outstanding lower corrosion property can be explained by the absence of halogen and sulfur in their molecules.^25^

### 3.3 Tribological properties

#### 3.3.1 Effect of additive concentration

**Fig. 4:**
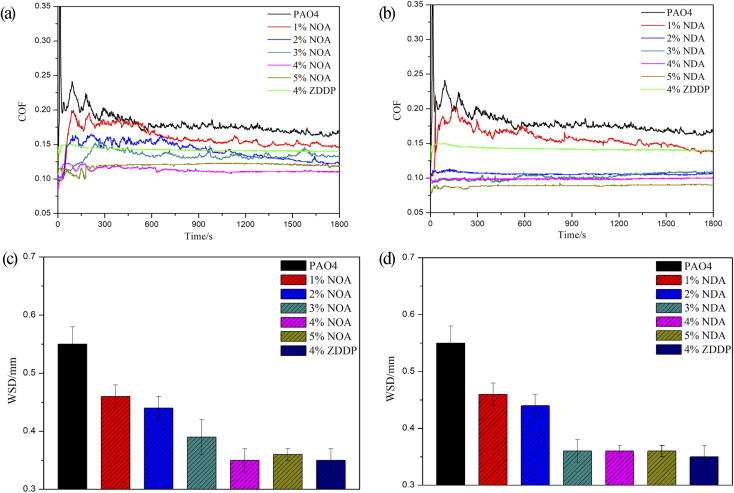
COF curves of (a) PAO4, PAO4 and NOA blends, 4% ZDDP and PAO4 blend; (b) PAO4, PAO4 and NDA blends, 4% ZDDP and PAO4 blend; WSD of upper ball surface after SRV test for (c) PAO4, PAO4 and NOA blends, 4% ZDDP and PAO4 blend; (d) PAO4, PAO4 and NDA blends, 4% ZDDP and PAO4 blend (with load of 100 N, frequency of 25 Hz, amplitude of 1 mm, temperature of 50 °C and time of 30 min).

The tribological properties of NOA and NDA in PAO4 were investigated by SRV (50 °C, 100 N, 25 Hz, 1 mm, 30 min) for steel/steel contact, as shown in Fig. 4. The coefficient of friction (COF) of PAO4 experienced a high fluctuation for the first 500 s sliding and subsequently dropped to approximately 0.150 with slight fluctuation. It can be observed that PAO4 with 1 wt% NOA or NDA could slightly reduce COF and wear scar diameter (WSD) compared to pure PAO4, indicating that the addition amount is not enough to provide good friction reduction performance. With the increase of additive concentration, the value of COF decreased. When the concentration of NOA was 4 wt%, the COF reached the lowest value of 0.103, which was approximately 31% reduction compared to pure base oil. The WSD of PAO4 with 4 wt% NOA was about 0.35 mm which was 36% lower than that of PAO4 base oil. For PAO4 and NDA blends, the COF was decreased to about 0.116 for PAO4 with 3 wt% NDA, and WSD was 0.36 mm. Further increase of the NDA concentration showed negligible effect on the tribological performance. The COF of PAO4 with 4 wt% ZDDP was about 0.142 and WSD was 0.35 mm. Compared with ZDDP, the friction reduction performance of PAO4 with 4 wt% NOA or NDA blends was greatly improved, but the anti-wear performance is similar. The above results demonstrated that the synthesized NOA and NDA were effective additives in terms of reducing COF and enhancing the wear protection performance of base oil.

#### 3.3.2 Effect of load

**Fig. 5:**
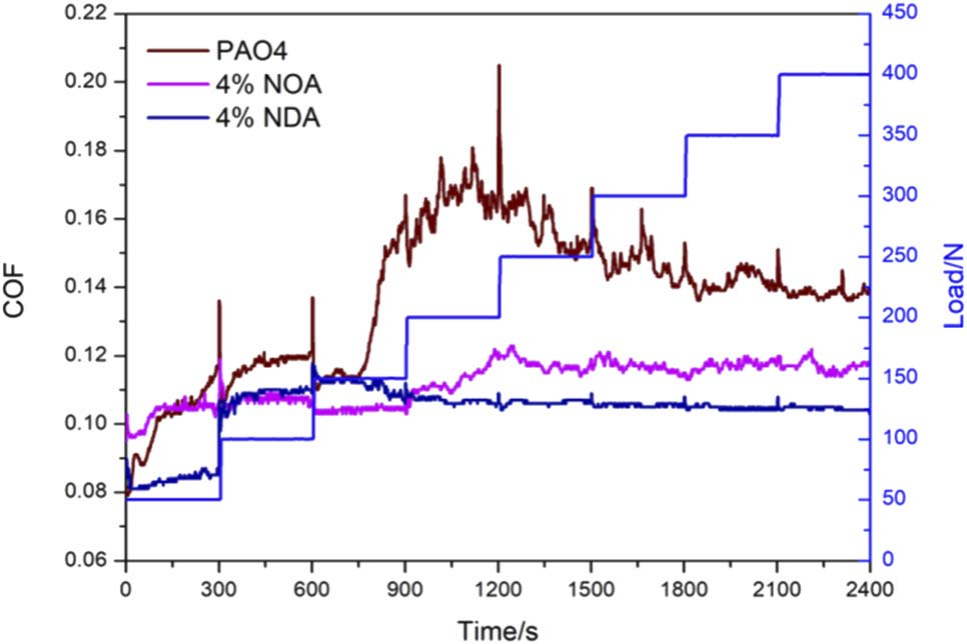
Evolution of COF with time of the PAO4, PAO4 and 4 wt% NOA blend, and PAO4 and 4 wt% NDA blends with different load (with frequency of 25 Hz, amplitude of 1 mm and temperature of 50 °C).

Fig. 5 displayed a load ramp test stepped from 50 N up to 400 N by the intervals of 50 N for PAO4, PAO4 and 4 wt% NOA blend, PAO4 and 4 wt% NDA blends. The COF of PAO4 base oil showed a large fluctuation with the increase of load. The COF of PAO4 with 4 wt% NOA blends exhibited a slight increase, and then keep almost stable (about 0.116 to 0.118) during the load from 250 N up to 400 N. The COF of PAO4 with 4 wt% NDA showed the most stable and the lowest value (about 0.104 to 0.106) when the load was from 100 N to 400 N. Compared with PAO4 and 4 wt% NOA blend, PAO4 and 4 wt% NDA blend showed lower COF under heavy load condition, suggesting the better load carrying property of NDA. The results can be attributed to the non-polar property of PAO molecules in that they do not easily adsorb on the metal interface. However, NOA or NDA ILs has polar functional groups and long alkyl chain length, which has an important positive effect on stability of the boundary lubricant film. In addition, the tribological performance is further improved by introducing more long alkyl chain in chemical structure of the ILs, since NDA as dicationic ILs has longer alkyl chain which is beneficial to the formation of boundary film.^26,27^ Thus, compared to PAO4, PAO4 and NOA blends, the maximum reduction in COF was achieved by PAO4 and NDA blend at high load conditions.

#### 3.3.3 Effect of frequency

**Fig. 6:**
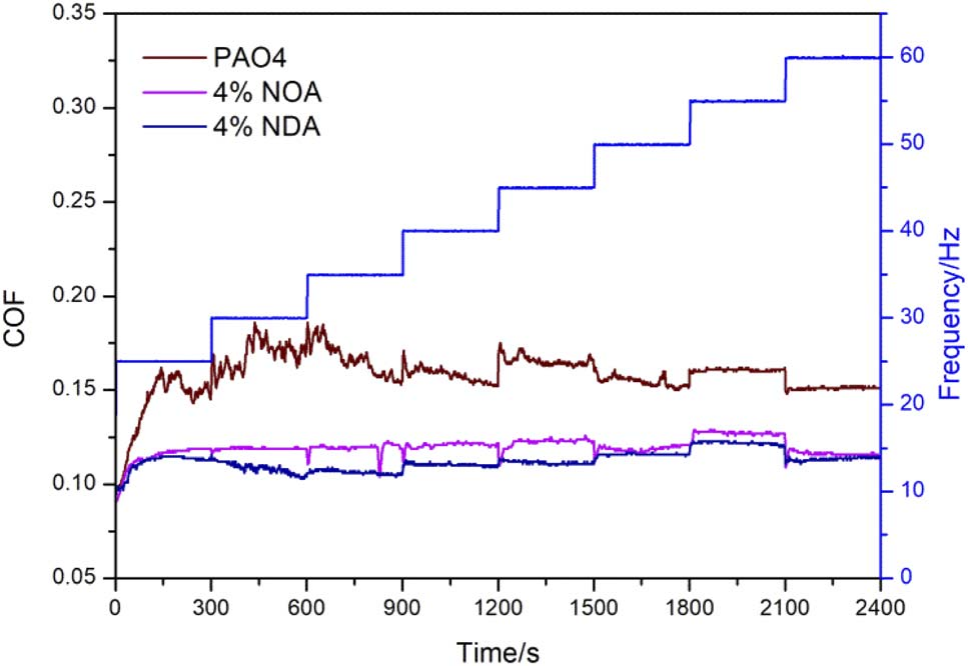
Evolution of COF with time of the IL and PAO4 blends with different frequency (with load of 100 N, amplitude of 1 mm, temperature of 50 °C).

Fig. 6 showed a frequency ramp test stepped from 25 Hz up to 60 Hz by 5 Hz intervals at a load of 100 N for pure PAO4, 4 wt% NOA and PAO4 blends, 4 wt% NDA and PAO4 blends. With the increase of frequency, the COF of pure PAO4 experienced a high fluctuation from 25 Hz to 35 Hz, and then kept at a relatively stable state. The COFs of PAO4 with ILs kept at slightly fluctuation from 25 Hz to 60 Hz and showed lower value compared to pure PAO4 during the whole test, implying that the NOA and NDA can effectively improve the tribological behavior of PAO4 base oil under different frequencies.

### 3.4 Worn surface analysis

**Fig. 7:**
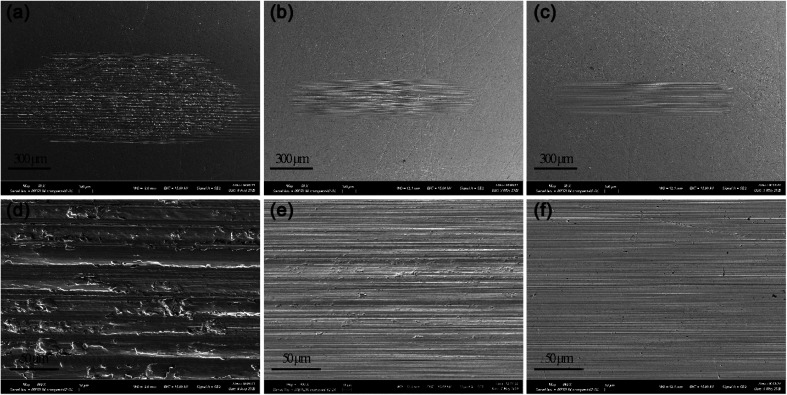
SEM micrographs of wear scars formed on the lower disc during the tribological test of PAO4 (a) and (d), 4 wt% NOA + PAO4 (b) and (e), and 4 wt% NDA + PAO4 (c) and (f).

SEM micrographs of wear scars after SRV test were shown in Fig. 7. The disc lubricated with PAO4 showed a wider wear scar with a lot of deep furrows, indicating the severe adhesive wear and plastic deformation of friction pairs. Worn surface lubricated by PAO4 with 4 wt% NOA was much smoother and shallower than pure base oil. PAO4 with NDA blends exhibited a similar image with NOA. The SEM micrographs of wear scars correlate well with the WSD results of PAO4 and ILs blends.

**Fig. 8:**
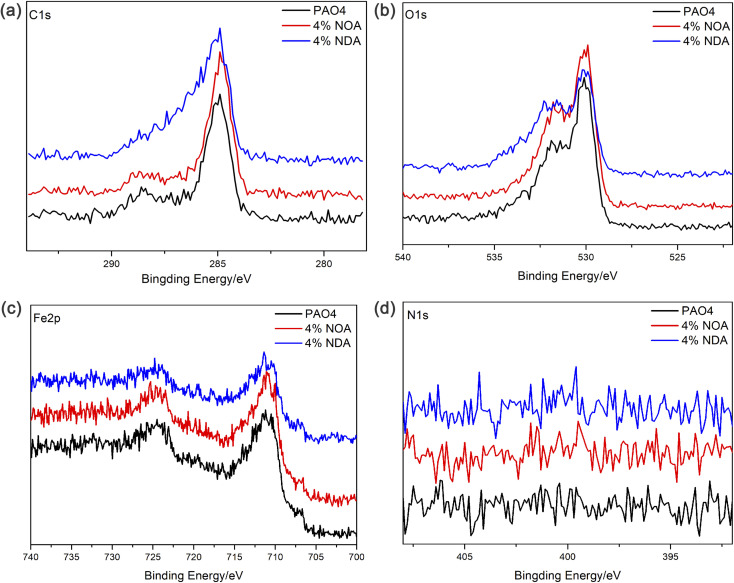
C1s, O1s, Fe2p and N1s XPS region scans for wear scars of different lubricants.

**Fig. 9:**
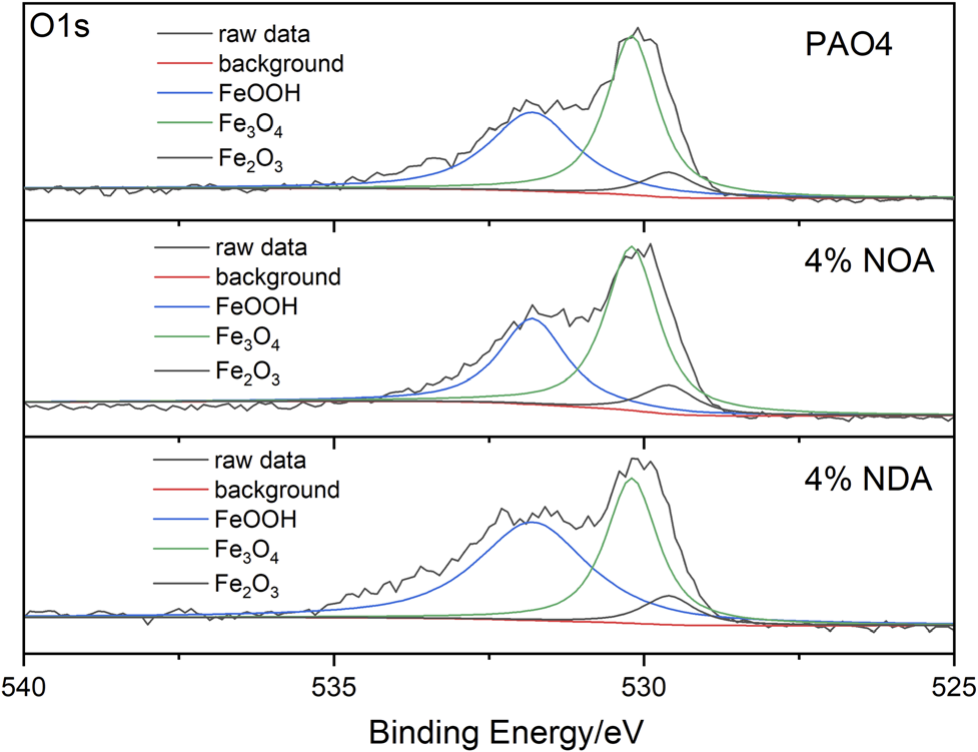
O1s XPS detailed spectras of lower disc wear scars lubricating by different lubricants.

XPS analysis of wear scars after SRV tests were carried out to investigate the chemical compositions of the worn surface after tribological tests. As shown in Fig. 8, C, O and Fe were abundant on the surface of wear scars. The binding energy of 284.8 eV of C1s can be assigned to organic carbon, which is probably due to the adsorption of the ILs. The binding energy of Fe2p appears at 710.7 eV and 724.3 eV are assigned to Fe_2_O_3_, Fe_3_O_4_ and FeOOH. As shown in Fig. 9, all the O1s spectra was assigned the 529.6, 530.2 and 531.7 eV, which corresponded to Fe_2_O_3_, Fe_3_O_4_ and FeOOH.^28^ However, N element was not detected on the wear scar surface, indicating the amine group didn't participate the tribochemical reaction. Based on the above XPS analysis, no fatty acid in the steel surface can be detected after tribological tests. It is believed that the physical adsorption of fatty acid plays an important role of the tribological performance.^29–31^ The physical adsorption of fatty acid provides a strong protection of the steel surface, which may be removed when we clean the surface after the test.

### 3.5 Anti-oxidation performance of ILs

**Fig. 10:**
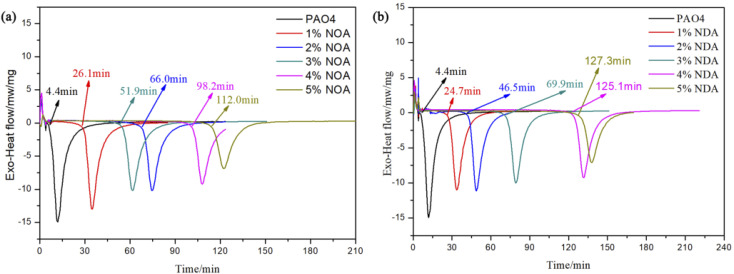
The PDSC results of (a) PAO4 and NOA blends and (b) PAO4 and NDA blends.

PDSC is a rapid and accurate technique to evaluate the oxidation stability of lubricants and has been utilized to study the oxidation stability of lubricants by many workers.^32–35^Fig. 10 displays the OIT results of different lubricants. It is observed that the OIT of pure PAO4 was merely 4.4 min, while a combination of PAO4 with NOA showed much higher oxidation stability. The OIT of PAO4 with 4 wt% NOA was 98.2 min which was almost 22 times higher than pure PAO4. PAO4 with 5 wt% NDA exhibited the highest OIT results of 127.3 min which is an almost 28 times increase compared to pure base oil demonstrating the outstanding antioxidation properties of the synthesized additives. It is worth noting that NDA produced higher OIT than NOA which suggests that NDA is more effective to improve the antioxidant stability of base oil. The outstanding antioxidation properties of ILs were probably due to the presence of alkyl aromatic amine group of cations.^14^

### 3.6 The lubricating and anti-oxidation mechanism

**Fig. 11:**
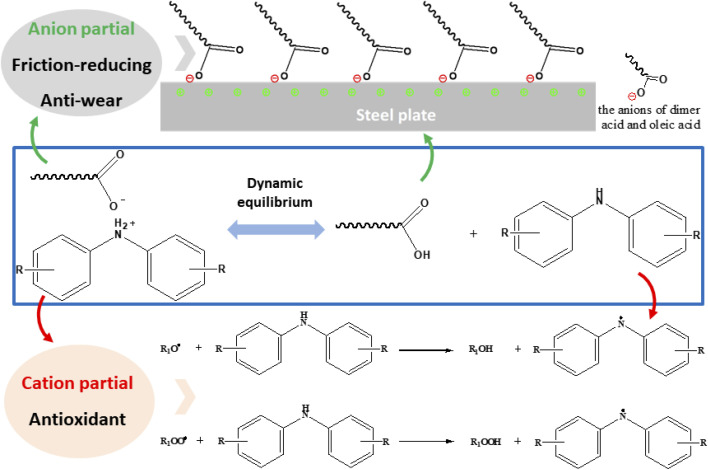
The lubricating and anti-oxidation mechanism of ILs additives.

Protic ILs NOA and NDA were synthesized by transferring a proton from acid to amine. This process involves a dynamic equilibrium between ILs and their free cations and anions and the interactions forces is much weaker than their aprotic counterparts, which made protic ILs are sensitive to the working conditions.^24^ During the sliding process, free carboxylate anions tend to adsorb onto the sliding steel surfaces and form a boundary lubrication film which will reduce the friction coefficient and protect metal surface from wear.^29^ In classical adsorption model for ILs, the ILs dissolved in the oil are attached to the metal surface by the polar groups of the molecules, and the long-chain hydrocarbons are dissolved in the oil, forming a multilayer matrix of friction improver molecules perpendicular to the metal surface.^36,37^ It is noteworthy that the ordered and dense films have fewer conformational defects and thus exhibit lower friction levels.^37^ The interfacial physical adsorption models are shown in Fig. 11, in which the green and red represent cations on surface and anions in additives respectively. Obviously, the adsorption phenomena of fatty acid on the surface of the steel disc may lead to the formation of a dense and ordered adsorption layer. In this paper, when PAO4 base oil film is not stable existence on the surface, the ILs contribute more to the formation of low-friction surface. Therefore, the physical adsorption of fatty acid plays an important role of the tribological performance in PAO4 base oil. The lubrication mechanism is similar to the ionic liquid described in the previous literature.^38^

In addition, when the lubricant was subjected to oxidation conditions, free amine parts are readily to react with free radicals to enhance the oxidation stability of the lubricants, as shown in Fig. 11. With the continually consumption of free cations and anions, the dynamic equilibrium was broken, more cations and anions will be released to work on the tribo-surface or the solvents. Their unique structure made them more suitable as anti-wear and anti-oxidation additives in various lubricants.

## 4. Conclusion

In this work, two multifunctional phosphorus-free protic ionic liquids were synthesized, characterized and evaluated, the main findings are as below:

(1) The synthesized ionic liquids were found completely miscible with various base oils including nonpolar hydrocarbon oils such as PAO4. It is mainly attributed to their long hydrocarbon chain of cation and anion which could generate high steric hindrance and effectively reduce the interactions between cation and anion.

(2) Due to the free of sulfur and halogen elements in molecular, both NOA and NDA showed no corrosion toward copper. In addition, the presence of alkyl aromatic amine group endows them excellent anti-oxidation performance in PAO4.

(3) When used as oil additives in PAO4, both NOA and NDA could effectively improve the tribological performance in a wide range of test conditions.

## Conflicts of interest

The authors declare no competing financial interest.

## Supplementary Material

RA-012-D2RA04006A-s001
