# Supplementary material for: Synthesis and investigation of phosphorus-free ionic liquids as multifunctional lubricating additives
Source: RSC Adv. 2022 Sep 5;12(39):25134–42. doi: 10.1039/d2ra04006a (PMC9443478; doi:10.1039/d2ra04006a)
Supplement: RA-012-D2RA04006A-s001 [file RA-012-D2RA04006A-s001.pdf]

Electronic Supplementary Material (ESI) for RSC Advances.
This journal is © The Royal Society of Chemistry 2022

Synthesis and Investigation of Phosphorus-free Ionic Liquids as Multifunctional Lubricating Additives

Huaigang Su[a, b, c],Qin Zhao[a], Yunlong Chen [a, c], Qilong Zhao [a, c], Cheng Jiang[a, c*], Wenjing Lou[a, c*]

[a] State Key Laboratory of Solid Lubrication, Lanzhou Institute of Chemical Physics, Chinese Academy of Sciences, Lanzhou 730000, P.R. China

[b] University of Chinese Academy of Sciences, Beijing 100049, P.R. China

[c] Qingdao Center of Resource Chemistry and New Materials, Qingdao 266100, P.R. China

1. ^{1}H-NMR of synthesized NOA and NDA

The ^{1}H-NMR spectra of starting materials, synthesized NOA and NDA were recorded on a Bruker AVANCE III 400 MHz spectrometer. $CDCl_3$ was used as solvent.

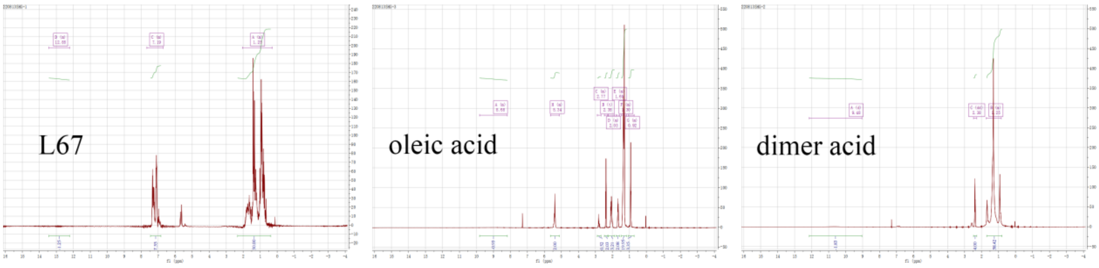

Figure S1 1H-NMR of starting materials

Nonylated diphenylamine (L67)

^{1}H-NMR (400 MHz, CDCl3) δ (ppm): 12.85 (m, 1H, -NH), 7.19 (m, 8H, arom), 0.38-2.32 (m, 30H, $-CH_2$/$-CH_3$)

Oleic acid

^{1}H NMR (400 MHz, CDCl3) δ (ppm): 12.85 (m, 1H, -COOH), 5.34 (m, 2H, -CH=CH-), 2.03-2.77 (m, 6H, $-CH_2$-CO-/$-CH_2$-C=C), 0.92-2.03 (m, 25H, $-CH_3$/$-CH_2$)

Dimer acid

^{1}H-NMR (400 MHz, CDCl3) δ (ppm): 9.48 (d, 2H, -COOH), 2.38 (dd, 4H, $-CH_2$-CO-), 0.69-1.79 (m, 58H, $-CH_3$/$-CH_2$)

NOA

^{1}H-NMR (400 MHz, $CDCl_3$) δ (ppm): 7.19 (m, 8H, arom), 5.46 (m, 2H, -CH=CH-), 2.36 (m, 2H, $-CH_2$-CO-), 0.48-2.18 (m, 59H, $-CH_3$/$-CH_2$)

Corresponding author: E-mail address: wjlou@licp.cas.cn and jiangcheng@licp.cas.cn

NDA

^{1}H-NMR (400 MHz, $CDCl_3$) δ (ppm): 7.14 (m, 16H, arom), 2.40 (m, 4H, $-CH_2$-CO-), 0.42-2.13 (m, 119H, $-CH_3/-CH_2$)

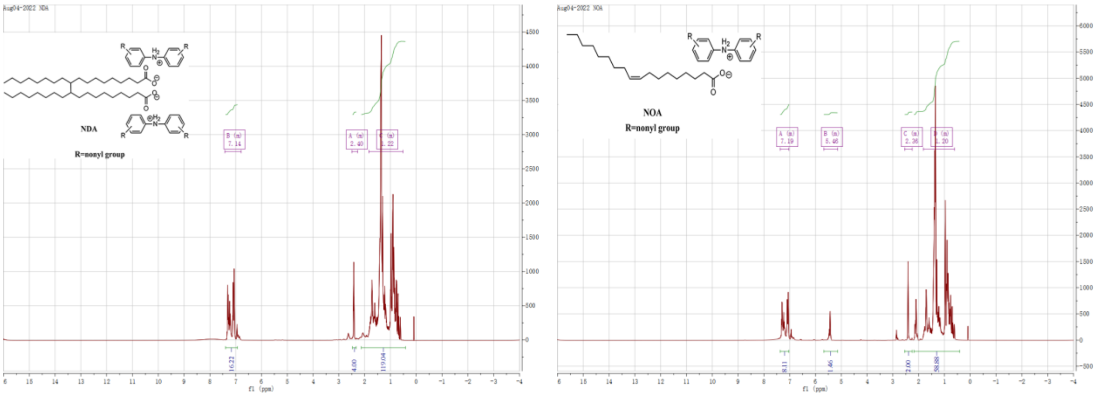

Figure S2 1H-NMR of synthesized ionic liquid

Due to the influence of hydrogen bond, the active hydrogen does not show obviously. Referring to ^{1}H-NMR of oleic acid and dimer acid, it confirmed the existence of NDA and NOA. The complex peak in δ=1-2 is due to the impurities from Nonylated diphenylamine (L67), because L67 is a commercial sale mixture.
